# A TCAD Study on High-Voltage Superjunction LDMOS with Variable-K Dielectric Trench

**DOI:** 10.3390/mi13060843

**Published:** 2022-05-28

**Authors:** Zhen Cao, Qi Sun, Hongwei Zhang, Qian Wang, Chuanfeng Ma, Licheng Jiao

**Affiliations:** School of Artificial Intelligence, Xidian University, Xi’an 710071, China; qi_sun0328@163.com (Q.S.); zhw2813532830@163.com (H.Z.); wangq_amber@163.com (Q.W.); machuanfeng1021@163.com (C.M.); lchjiao@mail.xidian.edu.cn (L.J.)

**Keywords:** superjunction, LDMOS, electric field modulation, dielectric RESURF, breakdown voltage

## Abstract

In this paper, a novel high voltage superjunction lateral double diffused MOSFETs (SJ-LDMOS) using a variable high permittivity (VHK) dielectric trench is presented. A relatively high HK dielectric is in the upper trench, which is connected with the drain electrode to suppress the high electric field (E-field) peak under the drain by the dielectric reduced surface field (RESURF) effect. In addition, a relatively low HK dielectric is at the bottom of the trench. On the one hand, the substrate is effectively depleted by a suitable HK dielectric layer, and the vertical depletion region of the substrate is greatly expanded. On the other hand, the overall vertical bulk E-field distribution is modulated by the E-field peaks generated at the position of varying K dielectric. A more uniform bulk E-field distribution is obtained for VHK SJ-LDMOS, leading to a high breakdown voltage (BV). Compared to the conventional SJ-LDMOS, the blocking voltage per micron of the drift region of VHK SJ-LDMOS has increased by 41.2%. Besides, compared with the SJ-LDMOS with a uniform-K, the BV of VHK SJ-LDMOS is improved by about 9.5%. The condition of the optimal range of the variable high permittivity is also presented. Meanwhile, the proposed VHK SJ-LDMOS has good conduction characteristics and heat dissipation

## 1. Introduction

Superjunction (SJ) power devices can achieve remarkable low on-resistance while achieving high breakdown voltage (BV), breaking the traditional MOSFET silicon limit [1,2]. Lateral double-diffused MOSFETs (LDMOS) using SJ technology (SJ-LDMOS) may be the key element in high voltage (700 V-class) power integrated circuits (PIC) [3,4]. For the purpose of avoiding costly SOI (silicon on insulator) and DI (dielectric isolation) substrate, the bulk silicon SJ-LDMOS with good heat dissipation was developed. To eliminate the substrate assisted depletion effect and improve the performance of SJ-LDMOS, several structures have been reported [5,6,7,8]. However, the performance of these devices is still affected by the curvature effect of the N+ drain diffusion and the uneven bulk electric field (E-field) distribution [9,10], which causes the problem that the BV of SJ-LDMOS is prone to saturation as the drift region length (L_D_) increases (L_D_ ≥ 40 μm). Thus, the blocking voltage per micron of the drift region (~13 V/μm) is far less than that of the theoretical value of the silicon drift region for charge-coupled structures (~20 V/μm) [11,12].

**Figure 1:**
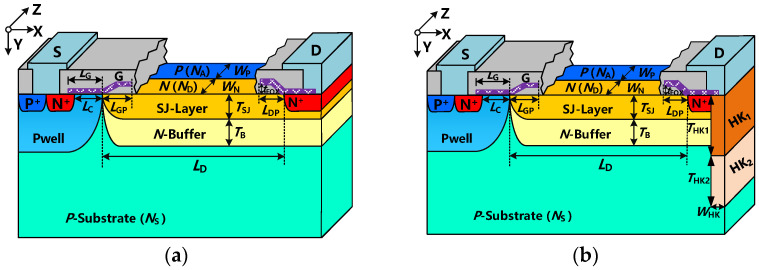
Three-dimensional view of (**a**) conventional SJ-LDMOS and (**b**) VHK SJ-LDMOS.

In order to optimize the bulk E-field distribution and further improve the trade-off between BV and R_on,sp_, we propose a novel SJ-LDMOS (Figure 1b) with a deep trench filled with variable high permittivity (VHK) dielectric under the N+ drain, to suppress the bulk peak E-Field around the edge of the drain diffusion and improve the overall E-field distributions. The main idea of the proposed VHK SJ-LDMOS is to further improve BV by the bulk E-field modulation provide b + -y the VHK dielectric trench. Simulation results show that the blocking voltage per micron of the drift region of VHK SJ-LDMOS has increased by 41.2%, compared with that of the conventional one. Besides, the proposed VHK SJ-LDMOS has good conduction characteristics and heat dissipation. The main physics models are applied to the Sentaurus TCAD simulation, including Mobility (DopingDep High Field satEnormal), EffectiveIntrinsicDensity (OldSlotboom), Recombination (SRH (DopingDep) and eAvalanche (CarrierTempDrive)). The criterion of breakdown is BreaCriteria {Current (Contact = “drain” Absval = 1.0 × 10^−7^ A)}. The main solving model is Coupled {Poisson Electron Hole}.

## 2. Device Structure and Mechanism

The schematic three-dimensional structure of the proposed VHK-SJ-LDMOS and the conventional SJ-LDMOS structure is shown in Figure 1. The main feature of the proposed VHK-SJ-LDMOS structure is with VHK dielectric trench under the N+ drain. According to the principle of dielectric reduced surface field (RESURF), the VHK dielectric layer is designed according to different concentrations of N^+^ drain, N-buffer layer and P-substrate. In this instance, Pb (Zr_x_Ti_1−x_) O_3_(PZT) is used to form the HK region due to its high enough permittivity without a complex process [13,14,15,16]. The process flow is the same as the conventional N-buffer SJ-LDMOS except for the implantation of the VHK trench under the drain region. A relatively high HK dielectric (HK_1_) in the upper trench, which is connected with the drain electrode to suppress the high E-field peak under the drain by the dielectric RESURF [17]. In addition, a relatively low HK dielectric (HK_2_) compared with the upper HK dielectric is at the bottom of the trench. In this paper, 3-D device simulations are conducted at room temperature. The key parameters of the optimized SJ-LDMOS structures used in the simulation are all listed in Table 1.

**Figure 2:**
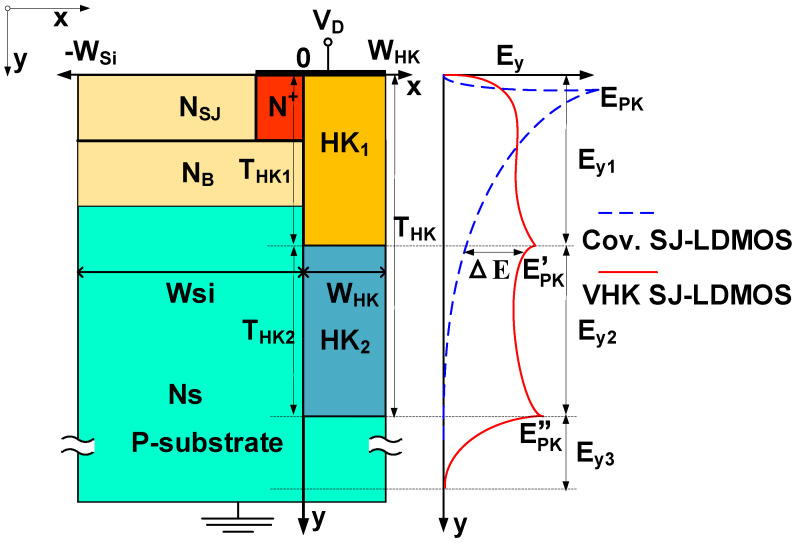
Schematic cross-section view and E-field component profiles of VHK LDMOS.

Figure 2 shows the schematic cross-section view and E-field component profiles of VHK SJ-LDMOS. In the off-state, the composite layer of HK and semiconductor under the drain jointly sustains the vertical bias voltage (V_R_) of the VHK SJ-LDMOS, which can be qualitatively considered as a semiconductor with an equivalent permittivity of (ε_Si_ + ε_HK_). The Variable-K pillar beside the substrate, the drain electrode/VHK/oxide/Si structure can be treated as a metal-insulator-semiconductor (MIS) capacitance [14,15], which makes it easier to deplete a higher N_S_ (allowing for a lower resistivity substrate) and a higher N_B_ by the assisted depletion effect to decrease R_ON,sp_.

According to the Poisson equation and dielectric RESURF [17,18,19,20], the vertical E-field component (E_Si,y_) is modified by the effective doping concentration Neff of semiconductor, which can be expressed as

(1) $$\frac{\partial E_{\mathrm{Si},y}}{\partial y}=\frac{{\mathrm{qN}}_{D}}{\varepsilon_{\mathrm{Si}}}-\frac{2V_{R}}{r_{y}^{2}}\frac{W_{\mathrm{HK}}}{W_{\mathrm{Si}}}\frac{\varepsilon_{\mathrm{HK}}}{\varepsilon_{\mathrm{Si}}}=\frac{{\mathrm{qN}}_{\mathrm{eff}}}{\varepsilon_{\mathrm{Si}}}$$

where r_y_ is the depth of the vertical depletion region. W_Si_ and W_HK_ are the widths of the semiconductor and HK dielectric, respectively. In order to obtain a uniform vertical electric field, HK trench layers with different dielectric constants are designed according to the semiconductor doping concentration under the drain. The doping concentration of the N+ pillar junction and the N-type buffer layer near the drain end is high, and there is a junction curvature effect, thus a relatively high dielectric layer is needed at the top of the trench to weaken the peak electric field under the drain. However, the doping concentration of the substrate is low, thus a relatively low HK dielectric is at the bottom of the trench. Additionally, because of the change of the K value, a new electric field peak appears to modulate the vertical electric field of the device, thereby achieving the purpose of optimizing the vertical electric field distribution of the device. Under the conditions of the avalanche breakdown, the theoretical BV of VHK SJ-LDMOS is obtained by the integral of the lateral and vertical E-field component as

(2) $$\mathrm{BV}\approx \mathrm{Min}\left[\int_{0}^{L_{D}}E_{\mathrm{Si},x}\mathrm{dx},\left(\int_{0}^{T_{\mathrm{HK}1}}\left|E_{\mathrm{Si},y1}\right|\mathrm{dy}+\int_{T_{\mathrm{HK}1}}^{T_{\mathrm{HK}}}\left|E_{\mathrm{Si},y2}\right|\mathrm{dy}+\int_{T_{\mathrm{HK}}}^{r_{y}}\left|E_{\mathrm{Si},y3}\right|\mathrm{dy}\right)\right]$$

## 3. Results and Discussion

### 3.1. Off-State Characteristics

**Figure 3:**
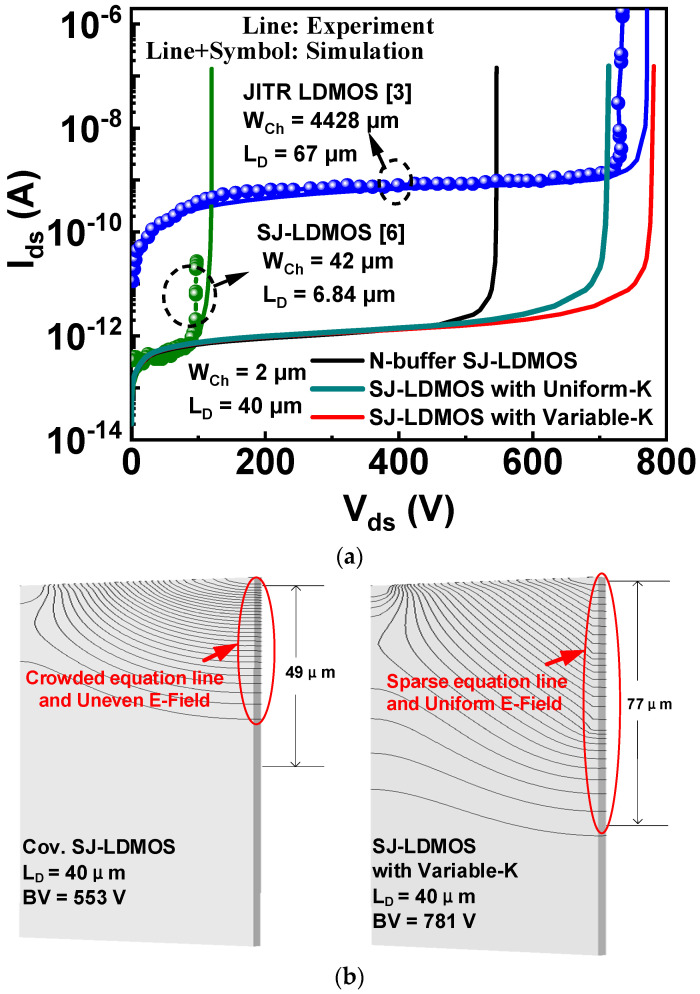
(**a**) Experimental and simulation breakdown characteristics for the conventional buffered SJ-LDMOS, VHK SJ-LDMOS, and Triple RESURF LDMOS. The experimental data were extracted from the experimental Ids-Vds curves in N-Buffer SJ-LDMOS and JITR LDMOS. (**b**) Equipotential contour plots of conventical SJ-LDMOS and VHK SJ-LDMOS (W_N_ = W_P_ = 1.0 µm, N_D_ = N_A_ = 5.0 × 10^16^ cm^−3^, L_SJ_ = 40 µm).

In Figure 3a, TCAD simulation is calibrated to experimental breakdown characteristics (Ids-Vds) data extracted from N-Buffer SJ-LDMOS [6] and Junction-Isolated Tiple RESURF (JITR) LDMOS [3]. With one set of self-consistent parameters, the TCAD simulation results and the experimental data are well matched. Figure 3a shows the simulated breakdown curve of VHK SJ-LDMOS and conventional SJ-LDMOS. Compared to conventional SJ-LDMOS (553 V), the BV of VHK SJ-LDMOS (781 V) is significantly improved by about 41.2% with a low leakage current. Besides, compared with the SJ-LDMOS with a uniform-K (713 V) [16], the BV of VHK SJ-LDMOS is improved by about 9.5%. This is due to the improved vertical E-field distribution of VHK TR LDMOS, which is modulated by the E-field peaks generated at the K variable position and the bottom of the VHK dielectric trench. Thus, the proposed VHK SJ-LDMOS can obtain a higher voltage than the conventional SJ-LDMOS and the SJ-LDMOS with a uniform-K. In Figure 3b, the equipotential contours of VHK SJ-LDMOS are more evenly spaced and the depletion area extends deeper (77 μm) than that of the conventional SJ-LDMOS (49 μm). The P-substrate is completely depleted by the VHK dielectric trench to obtain a much higher BV at the same drift length.

**Figure 4:**
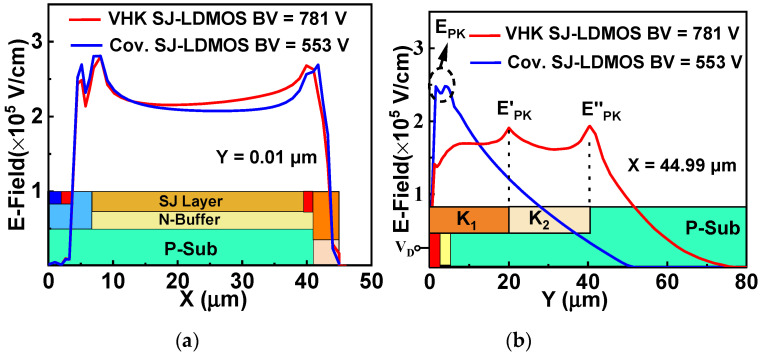
(**a**) Lateral electric field distributions for the conventional SJ-LDMOS and VHK SJ-LDMOS in the middle of the N-pillar along the X-direction, (**b**) vertical electric field distributions for the two devices perpendicular to the drain at X = 44.99 µm.

In Figure 4a, when the lateral E-field is optimized to a very uniform degree by RESURF and field modulation technology, etc., the BV does not increase as the LD increases. At this time, in order to further improve BV, the vertical bulk E-field of the device needs further optimization. Figure 4b shows the vertical E-field distribution near the drain end (at X = 44.99 μm). The high E-field peak (E_PK_) generated by the curvature effect of the N^+^ drain diffusion is effectively suppressed by the relatively high HK dielectric layer in the upper trench. Meanwhile, new E-field peaks (E′_PK_ and E″_PK_) brought about by varying HK dielectric greatly improve the vertical E-field distributions.

**Figure 5:**
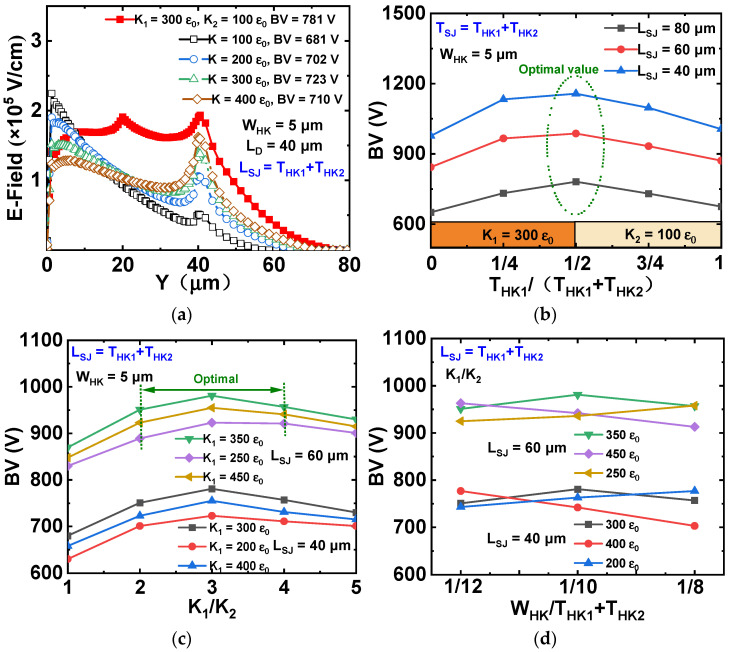
(**a**) Dependences of vertical E-field and BV on different K values, (**b**) dependences of BV on different ratios T_HK1_ to (T_HK1_ + T_HK2_) with three different L_SJ_ (40 μm, 60 μm and 80 μm), (**c**) dependences of BV on different ratios of K_1_ to K_2_, (**d**) dependences of BV on different ratios of W_HK_ to (T_HK1_ + T_HK2_) for VHK SJ-LDMOS.

Figure 5a shows the dependence of vertical E-field distributions and BV on different K values. Compared to a uniform HK dielectric-filled trench, VHK structure with suitable K combination (K_1_ = 300 ε_0_ and K_2_ = 100 ε_0_) has more uniform bulk E-field distributions, thus a higher BV is obtained. The dependences of BV on different ratios of T_HK1_ to (T_HK1_ + T_HK2_) with three different L_SJ_ (40 μm, 60 μm and 80 μm) are shown in Figure 5b. For a certain W_HK_, K_1_ and K_2_, there is an optimal value of T_HK1_/(T_HK1_ + T_HK2_) = 1/2 to achieve optimal performance. The dependences of BV on different ratios of K_1_ to K_2_ with different K_1_ values are shown in Figure 5c. At the condition of the optimal range of K_1_/ K_2_, a higher BV is obtained. The optimal range is 200 < K < 400. Pb (Zr_x_Ti_1−x_) O_3_ (PZT) is a good candidate to realize the high relative permittivity because PZT is easy to etch and can achieve the optimal K values without complex processing. Figure 5d shows the dependences of BV on different ratios of W_HK_ to (T_HK1_ + T_HK2_) at different K values and L_SJ_. The optimal ratios of W_HK_ to (T_HK1_ + T_HK2_) of VHK SJ-LDMOS to ensure that the vertical depletion depth spreading in the substrate, to obtain an optimized BV by the reshaping effect of the HK trench enhanced the vertical electric field strength in the substrate.

### 3.2. ON-State Characteristics

**Figure 6:**
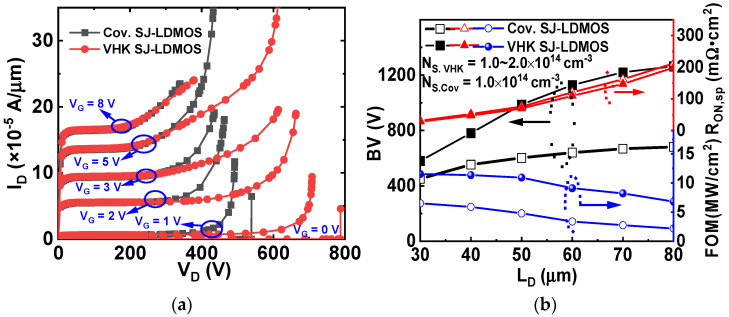
(**a**) Output characteristics for VHK SJ-LDMOS and conventional SJ-LDMOS, (**b**) dependences of BV, R_ON,sp_ and FOM on L_D_ for VHK SJ-LDMOS and conventional SJ-LDMOS.

Figure 6a shows the output characteristics of the conventional SJ-LDMOS and VHK-MOSFET. The V_TH_ (threshold voltage) are both about 2.0 V. At different gate voltages, V_G_, the VHK-MOSFET has a higher BV than that of the conventional SJ-LDMOS due to the uniform electric field distribution. The dependences of BV, R_ON,sp_ and figure-of-merit (FOM = BV^2^/R_ON,sp_) on LD for VHK SJ-LDMOS and the conventional SJ-LDMOS are shown in Figure 6b. It is found that the BV of VHK SJ-LDMOS increases faster and saturates at a longer L_D_ as the L_D_ increases (BV > 700 V at L_D_ = 40 μm).

**Figure 7:**
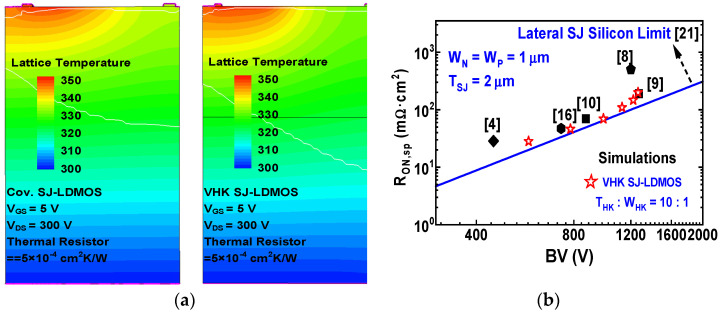
(**a**) Temperature distributions in the conventional SJ-DMOS and VHK SJ-LDMOS, (**b**) comparison of R_on,sp_ versus BV for VHK SJ-LDMOS and other exiting technologies.

Temperature distributions in the conventional SJ-LDMOS and VHK SJ-LDMOS are shown in Figure 7a, by S-device simulation. For temperature distribution simulations with the thermodynamic transport model, a thermal contact that coincides with a substrate electrode is defined. The keyword hydrodynamic (eTemperature) is specified in the global physics section to activate the hydrodynamic model in TCAD simulation. For the thermal contact, a temperature (300 K) is declared. The contact is attached by a thermal resistor with value = 5 × 10^−4^ cm^2^ K/W. A non-zero thermal resistance may be used to emulate heat exchanges of the device with the outside environment. It can be seen from the results that although the high current leads to the temperature of the two devices increasing, the two devices can still work normally with good ruggedness. Figure 7b shows the R_on,sp_ versus BV for VHK SJ-LDMOS and other existing SJ-LDMOS. As can be seen, VHK SJ-LDMOS exhibits better performance at the BV region (400–1200 V), which is close to the lateral SJ silicon limit under the optimized conditions [21].

**Figure 8:**
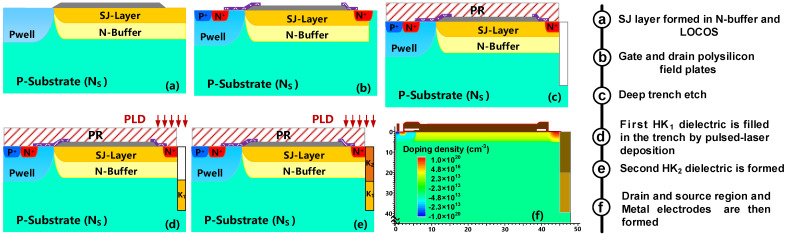
Key process steps for fabricating the proposed VHK SJ-LDMOS.

A feasible fabrication process of the VHK SJ-LDMOS is exhibited in Figure 8. The proposed VHK SJ-LDMOS fabrication starts with a P-type substrate material (100). In Figure 8a, first, an N-type buffer well and a P-well are formed by ion implantation, then the superjunction layer is formed in the N-type well, and the field oxide layer is formed by local oxidation of silicon (LOCOS) process. Next, source and drain are formed by N^+^ and P+ type ion implantation, respectively. A gate oxide layer is then grown, and polysilicon is deposited and etched to form a gate electrode, a gate field plate and a drain field plate, as shown in Figure 8b. In Figure 8c, a certain aspect ratio trench is then formed by the trench etch process. A dry oxidation is implemented to obtain a thin SiO_2_ buffer layer at the HK/Silicon interface. Then, PZT is deposited into the trenches by pulsed-laser deposition (PLD) technique, realizing different K values by different annealing temperature technology [22], as shown in Figure 8d,e. Finally, the passivation layer is deposited and then the electrodes of the device are further formed by depositing and etching metal. Based on this process, the proposed device is simulated using SPROCESS as shown in Figure 8f [23]. The key parameters are all listed in Table 2.

## 4. Conclusions

In conclusion, a new SJ-LDMOS with a variable high permittivity dielectric trench is presented in this paper. Based on the principle of dielectric RESURF, the VHK dielectric layer is designed according to different concentrations of N+ drain, N-buffer layer and P-substrate. The vertical bulk electric field of SJ-LDMOS is improved by the electric field modulation effect of the VHK trench. The results obtained by simulation show that the vertical electric field distribution is effectively enhanced, compared with conventional SJ-LDMOS, the blocking voltage per micron of the drift region of VHK SJ-LDMOS is increased by 41.2% with the same drift length, and the trade-off between BV and R_on,sp_ of VHK SJ-LDMOS is close to the lateral SJ silicon limit with good conduction characteristics and heat dissipation.

## Figures and Tables

**Table 1:** Device parameters in the simulation.

Symbol	Description	VHK SJ-LDMOS	Cov. SJ-LDMOS
W_N_, W_P_	N, P drift width (μm)	1.0	1.0
T_SJ_	SJ layer thickness (μm)	2.0	2.0
T_B_	Buffer layer thickness (μm)	3.0	3.0
L_GP_, L_DP_	Gate, drain poly FP length (μm)	3.0, 2.0	3.0, 2.0
t_OX_, t_FOX_	Gate, field oxide thickness (μm)	0.04, 0.8	0.04, 0.8
L_C_	Channel length (μm)	1.5	1.5
L_D_	Drift length (μm)	30–90	30–90
N_S_	Substrate doping (cm^−3^)	1.0–3.0 × 10^14^	1.0–2.0 × 10^14^
N_D_, N_A_	N, P drift doping (cm^−3^)	5.0 × 10^16^	5.0 × 10^16^
N_B_	Buffer layer doping (cm^−3^)	4.0 × 10^15^	3.0 × 10^15^
K	K dielectric values (ε_K_/ε_0_)	PZT (100–400)	--
T_HK_	HK trench depth (μm)	30–50	–
W_HK_	HK trench width (μm)	5–8	–

**Table 2:** Key parameters in the process.

Step	Region	Mask (μm)	Process	Recipe
(a)	P-Sub	/	Start	B/90 Ω·cm/<100>
	N-buffer	4.0–48.0	Implantation	A_S_/5 × 10^12^ cm^−2^/100 KeV/7°
	P-well	0–4.0	Implantation	B/7 × 10^13^ cm^−2^/150 KeV/7°
	N-pillar	4.0–48.0	Implantation	A_S_/5 × 10^13^ cm^−2^/80 KeV/7°
	P-pillar	4.0–48.0	Implantation	B/5 × 10^13^ cm^−2^/80 KeV/7°
	LOCOS	4.0–44.0	LPCVD SiO_2_	425 °C, Gas: SiH_4_ + O_2_/ 500 nm
(b)	Gate Oxide	1.5–5.0	Deposit and etch	SiO_2_/0.04 μm
	Poly-Si	1.5–5.0	Deposit and etch	PolySilicon/ 0.3 μm
	N^+^	1.0–2.0	Implantation	As/5 × 10^15^ cm^−3^/80 KeV/7°
	P^+^	0–1.0	Implantation	B/2 × 10^15^ cm^−3^/80 KeV/7°
(c)	Trench	44.0–48.0	Etch	Si/30 min/40 μm
	Thin Oxide	/	Deposit and etch	SiO_2_/0.04 μm
(d)	HK_2_	44.0–48.0	Deposit and etch	HK/20 μm/(permittivity)100 ɛ_0_
(e)	HK_1_	44.0–48.0	Deposit and etch	HK/20 μm/(permittivity)300 ɛ_0_
(f)	SiO_2_	0–0.5, 1.5–44.0	Deposit and etch	SiO_2_/1.0 μm
	Metal	0–3.0, 42.0–48.0	Deposit and etch	Al/0.4 μm

## Data Availability

Not applicable.
